# Constitutional mismatch repair deficiency: a case on a commonly misinterpreted mutation in colon cancer

**DOI:** 10.1007/s12328-024-02015-9

**Published:** 2024-08-02

**Authors:** King C, Edwards H, Thompson E, Abdelmasseh M, Cuaranta A, Pacioles A, Sanabria J

**Affiliations:** 1 Marshall University School of Medicine (MUSOM), Huntington, USA; 2 Department of Surgery at MUSOM, Huntington, USA; 3 Marshall Institute for Interdisciplinary Research (MIIR), Huntington, USA; 4 Department of Medicine at MUSOM, Huntington, WV USA

**Keywords:** Mismatch repair, Colorectal cancer, Lynch syndrome, HNPCC

## Abstract

It is estimated that 153,020 cases of CRC per year, with an increase in diagnoses in younger patients. We present a case of a female with an early presentation of Lynch Syndrome and CRC, who, on her third malignant presentation, was re-diagnosed as a constitutional mismatch repair deficiency.

## Introduction

**Fig. 1:**
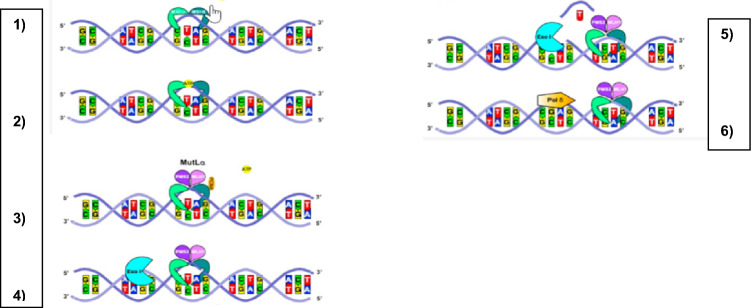
*Repair sequence in DNA mismatch.* 1) MutSα recognizes and binds a DNA mismatch. 2) MutSα-ATP/dependant conformational change. 3) Recruitment of PCNA. 4) PMS2 breaks a single strand and recruits Exo1. 5) Exo1 removes mismatch base/segment. 6) Polymerase δ synthetase the missing base/segment. These sequences, when abnormal, as in the patient of discussion, result in accumulation of mutations further increasing the risk of neoplasms

It is estimated 153,020 new cases of colorectal cancer (CRC) will be diagnosed in the US in 2023 [1]. CRC occurs from an accumulation of sporadic mutations; nevertheless, 5–10% arise from inherited mutations that present as hereditary cancer syndromes. Hereditary non-polyposis colorectal cancer (HNPCC) incidence continues to be on the rise and is typically associated with the Lynch syndrome (LS), a heterogenous autosomal dominant mutation in a mismatch repair (MMR) gene found on chromosome 2, 3, 5, or 7. Up to 82% of individuals with these mutations develop one of the closely associated cancers within their lifetime [7]. In replicating cells, frequent point errors during DNA duplication occur, but they are usually repaired by MMR gene proteins (Fig. 1). When these enzymes are dysfunctional, cells become unstable as they accumulate point mutations, in a process referred to as microsatellite instability (MSI). Microsatellites can be defined as short, repeated DNA sequences in our cells where the redundancy in DNA causes polymerase slippage during replication [2]. These sections are polymorphic with a wide genome variation where repeated bases are exceptionally prone to mutation. Although most of these microsatellite sections are spliced out during RNA processing, some hold essential functions in gene regulation, explaining further how their instability leads to unchecked growth modulation.

Mutations associated with the LS are seen commonly in MMR genes MLH1, MSH2, MSH6, or PMS2, predisposing patients to an earlier age malignancy. In contrast, Constitutional Mismatch Repair Deficiency (CMMR-D) syndrome is an autosomal recessive disease resulting from germline biallelic mutations in a DNA mismatch repair genes [3]. The former (LS) predisposes individuals to cancers such as colorectal, endometrial, and other tumors of endodermal origin presenting in the fourth decade of life. Despite the similarities, CMMR-D has an earlier onset of expression, with subjects developing malignancies earlier in their life, associated with hematologic and brain neoplasms. The diagnosis of LS can be suspected using the Amsterdam criteria upon patient presentation (Table 1) [4]. The diagnosis of CMMR-D does not have clearly identifiable criteria or protocol, likely contributing to the poor prognosis associated with this syndrome. To this note, the European Consort “Care for CMMR-D’ suggests a three-point system used to gage the likelihood of CMMR-D in pediatric/young adult patients. Points are correlated to a tumor’s specificity over-represented in CMMR-D population, and family history (Table 2) [5]. This proposition provides a guideline to follow, and a hope of identifying diseased individuals prior to the terminal development of their neoplasms. This may also allow for early surveillance and counseling, ideally ameliorating poor outcomes.

**Table 1:** Amsterdam criteria for the diagnosis of hereditary non-polyposis CRC (HNPCC) ^(5)^

Revised Amsterdam Il criteria and Bethesda guidelines for the clinical diagnosis of Lynch syndrome
Amsterdam II revised criteria: all of the following criteria should be met:
• Three or more blood relatives with a Lynch-related cancer (CRC, endometrial, small bowel, ureter, or renal pelvis)
• One relative must be a first-degree relative of the other two
• One or more cancer cases diagnosed before the age of 50 years
• Two or more successive generations affected
• Familial adenomatous polyposis excluded in CRC
• Tumor diagnosis confirmed by histopatholoqic examination
Revised Bethesda Guidelines:At least one of the following criteria should be met:
• CRC or endometrial cancer diagnosed before the age if 50 years
• Synchronous or metachronous CRC or other LS-associated tumors, regardless of age
• Colorectal cancer with MSl-high-associated morphologic features (Crohn-like lymphocytic reaction, mucinous/signet cell differentiation, or medullary growth pattern) in a patient younger than 60 years
• CRC in a patient with one or more first-degree relatives with an LS-associated tumor, with one of the cancers being diagnosed before the age of 50 years
• CRC is diagnosed in two or more first- or second-degree relatives with LS-associated tumors, regardless of age

*CRC* colorectal cancer, *LS* Lynch syndrome, *MSl* micro-satellite instability

**Table 2:** Sign and symptom-based scoring system for assessing and gaging likelihood of CMMR-d in pediatric/young populations [5]

Indication for CMMR-D Testing in a Patient With Cancer	3 Points
Malignancies and premalignancies: 1 is mandatory; if >1 is present in the patient, add the points:	
• Carcinoma from the LS spectrum^a^ at age <25 y	3 points
• Multiple bowel adenomas at age <25 y and absence of APC/MUTYH mutations or a single high-grade dysplasia adenoma at age <25 y	3 points
• World Health Organization grade Ill or IV glioma at age <25 y NHL of T-cell lineage or sPNET at age < 18 y	2 points
• Any malignancy at age <18 y	2 points
Additional features: Optional; if >1 of the following is present, add the points	I point
• Clinical sign of NF-I or ≥2 hyperpigmented or hypopigmented skin alterations ≥ 1 cm in the patient	2points
• Diagnosis of LS in a first-degree or second-degree relative	2points
• Carcinoma from LS spectrum^a^ before the age of 60 in first-degree, second-degree, or third-degree relative	1 point
• A sibling with carcinoma from the LS spectrum,^a^ high-grade glioma, SPNET, or NHL	2points
• A sibling with any type of childhood malignancy	1 point
• Multiple pilomatricomas in the patient	2points
• 1 pilomatricoma in the patient	1 point
• Agenesis of the corpus callosum or non—therapy-induced cavernoma in the patient	1 point
• Consanguineous parents	1 point
• Deficiency or reduced levels of immunoglobulin G 2/4 or immunoglobulin A	1 point

NHL non-Hodgkin lymphoma, SPNET supratentorial primitive neuroectodermal tumors

^a^Colorectal, endometrial, small bowel, ureter, renal pelvis, biliary tract, stomach, bladder carcinoma

## Case presentation

**Fig. 2:**
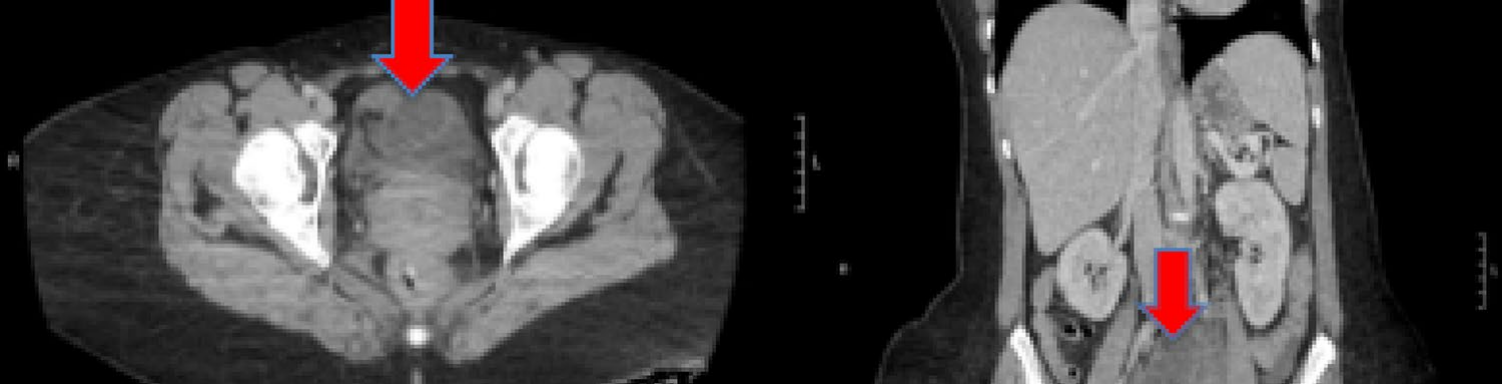
*CT scan of the abdomen with IV contrast.* It showed an annular lesion in the Jejunum (Image on the left with the red arrow at transition point), signaling as the cause of SBO (proximal dilation of SB loops as pointed by the arrow at the right image)

A 39-year-old female presented with a complaint of abdominal pain for 12 days associated with nausea and vomiting. CT scan of the abdomen revealed an annular constricting lesion in the jejunum, with fecalization of the proximal to the narrowing small-bowel segments (Fig. 2). Her past medical history includes the diagnosis of stage III right-sided node-positive colon cancer at age 14, treated with right hemicolectomy and diverting ileostomy**.** Following an uneventfully recovery, the patient had neoadjuvant therapy (5-flourouracil, leucovorin, and levamisole). At age 24, the patient had genetic testing done, which revealed a mutation in MSH6, a mismatch repair gene, consistent with the diagnosis of LS. A year later, at follow-up, she was diagnosed with rectal carcinoma, for which a left hemicolectomy was performed. **At this time, 5-flourouracil and oxaliplatin (FOLFOX) was used as adjuvant chemotherapy.** Other medical history includes bipolar disorder, ADHD, GERD, 0.5 pack/day smoking history from 2007–2014, alcohol consumption of 1 drink/day, and anemia. Family history is significant for LS on her parents, exemplifying its autosomal dominant inheritance pattern.

**Fig. 3:**
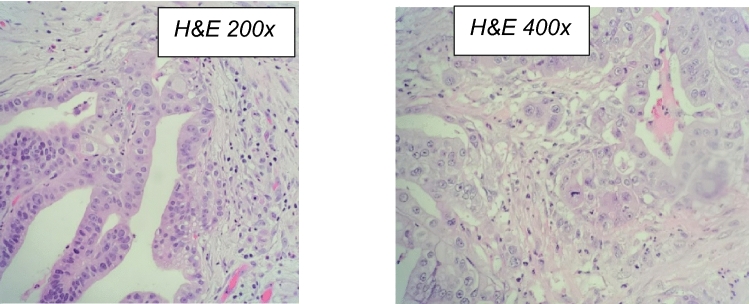
*Small-bowel mass following small-bowel resection:*- Adenocarcinoma, G2, moderately differentiated, 5.5 cm in greatest dimension, invading through the muscularis propria into the subserosa.- Lymphovascular invasion present.- All margins negative for invasive carcinoma

A CT scan of her small-bowel follow-through revealed an annular jejunal mass as the cause of her obstruction (Fig. 2). It was not clear if this was a primary or recurrent disease. In surgery, significant adhesiolysis was performed with a resection of 30 cm of jejunum containing the mass. GI continuity was established by primary jejuno-jejunal anastomosis. The patient recovered uneventfully. Pathology report of resected bowel showed a primary jejunal adenocarcinoma, described as moderately differentiated Grade 2, with a greatest dimension of 5.5 cm, invading through the muscularis propria up to the subserosa with lympho-vascular invasion (Fig. 3). All margins were negative for invasive carcinoma. Due to the higher risk for recurrence, oral adjuvant chemotherapy was used in addition to excisional procedures **(capecitabine plus oxaliplatin**).

Furthermore, a lobular enhancing midline structure in the pelvis was noted to be abnormal, slightly larger when compared to prior CT scans, and indicative of a fibroid uterus. CT scan of the pelvis revealed numerous T2-enhancing leiomyomas of the uterus. Genetic testing revealed a biallelic germline mutation of the MSH6 gene and MUTYH gene, consistent with a diagnosis of CMMR-D. To date, the patient works full-time, has negative hematologic and brain screenings, and continues to follow surveillance protocol.

## Discussion

**Fig. 4:**
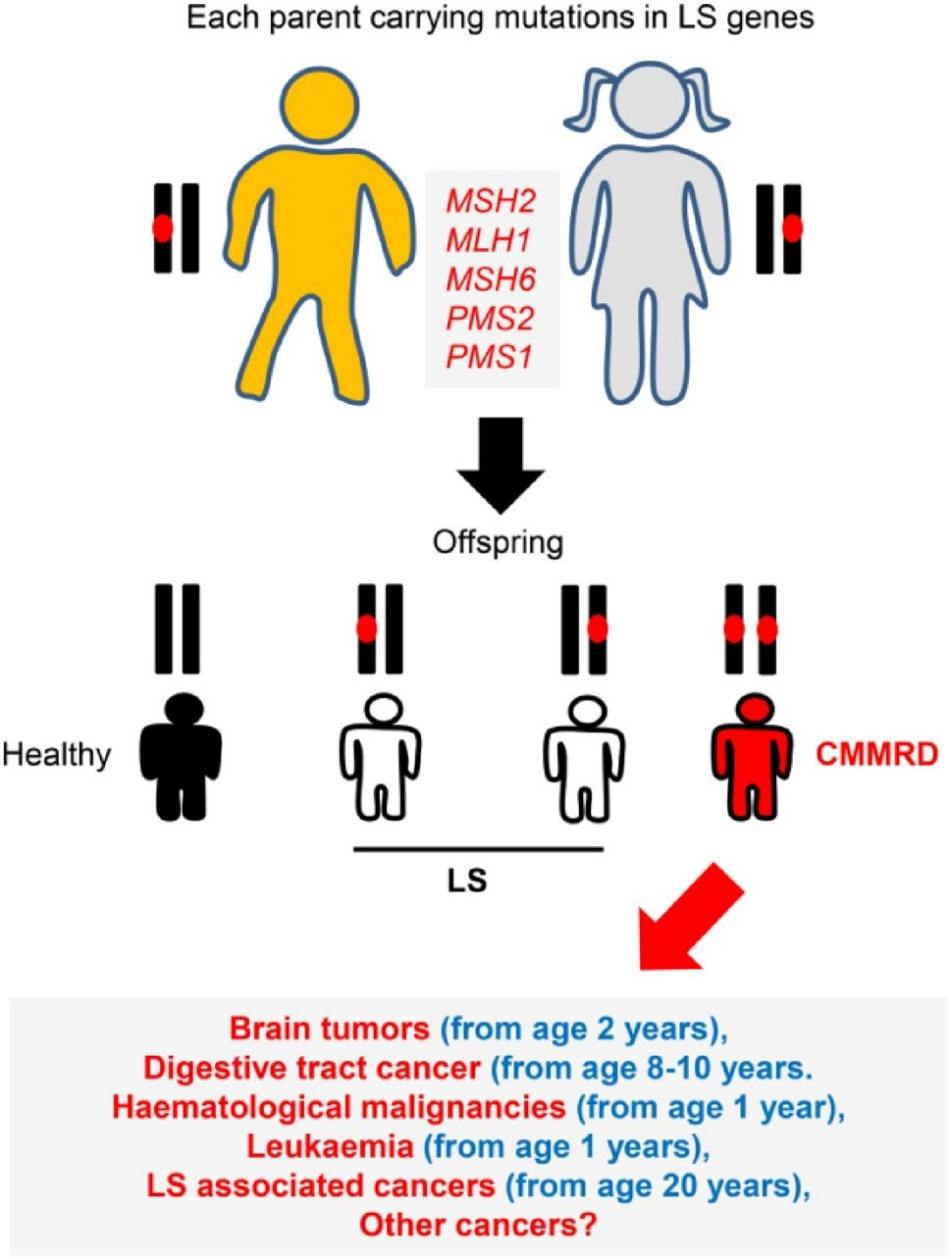
*Genetic transmission of MMR mutations*. To acquire HNPCC, recipient must receive one mutated copy of AD MMR mutation. CMMR-D offspring require two copies of mutated gene as they are inherited in AR fashion. The patient at discussion has parents that must both contain mutations in repair genes to have an established diagnosis of CMMR-D*.* [3]

To distinguish LS from the less common familial adenomatous polyposis, the term *hereditary nonpolyposis colon cancer* (HNPCC) was coined, and for many years the terms Lynch syndrome and HNPCC were used interchangeably. Earlier diagnosis of LS with high penetrance may have explained the history of early-onset CRC in the presented patient. To capture more families with hereditary cancer, the Amsterdam II Criteria was developed. These were based on the same criteria as Amsterdam I but were expanded to include several tumors associated with HNPCC in addition to CRC. Currently, the term HNPCC has been discouraged, because these patients do, in fact, make polyps, although not as many as is typical in Lynch syndrome [6]. Lynch syndrome must be distinguished from other familial adenomatous polyposis syndromes. The term *familial colorectal cancer type X* refers to the nearly 40% of patients who satisfy the criteria for Amsterdam I but do not have a genetic profile consistent with Lynch syndrome [6]. To date, however, a specific coding defect has not been identified to differentiate familial CRC type X at-risk family members by formal genetic testing. Nevertheless, further genetic testing in the presented patient revealed not only one mutated copy of her MSH6 MMR gene but an additional mutation on the opposing allele, establishing the diagnosis of CMMR-D. The most common initial approach uses immuno- histochemistry or microsatellite instability testing. Based on the results, patients can then be appropriately triaged and tested for formal germline defects. Mutated copies need to be passed on through meiosis, therefore each parent consequently must have been a carrier (Fig. 4).

CMMR-D is a rare syndrome with an incidence of 1/million patients [3]. Its infrequent occurrence has created a lack of awareness and often goes unnoticed until cancer occurrence and progression are diagnosed in young patients. Frequently associated neoplasms include colon (70%), brain (70%), small bowel (10%), and lymphoma/ leukemia (20–40%). In addition, patients with biallelic mutations in MSH6 have a significantly higher incidence of brain tumors manifested mainly in the first 10 years of life when compared to other mutations [6, 7]. MUTYH-associated polyposis syndrome (MAP) can be characterized as an autosomal recessive germline mutation in the MUTYH base excision repair gene and leads to an increased risk for gastrointestinal cancers [5]. Multiple adenomas can be found in the digestive tube from accumulated mutations leading to CRC. Although, the patient has not been diagnosed with MAP, this specific MUTYH mutation was confirmed in her genetic testing, placing her at risk for CRC. The former mutation confers a 28-fold increase in their likelihood of developing CRC [5, 8]. Currently, there are no absolute treatment options for those with CMMR-D. Prevention by continuous surveillance for early disease detection remains the best measure to implement proper treatment for an increase in lifespan with improved quality of life.

CRC presenting early in life should raise clinical suspicion of CMMR-D, especially those associated with brain, hematologic, and endodermal origin. Detailed exploration of family history and sibling presentation can be beneficial. Assessment in the history of pediatric/young adult malignancies may provide a score guiding indications for early surveillance and genetic testing in those with characteristics of CMMR-D. Once the diagnosis of CMMR-D is established, we recommend a multidisciplinary oncologic team to follow the patient with emphasis in the annual screenings of the GI, endometrium, and urinary tract. Blood panel and brain MRI should be done every 6 months.

## Conclusion

Subjects with an early-age diagnosis of CRC should undergo genetic testing or tissue analysis for MSI. If the diagnosis is established of LS or CMMR-D, annual follow-up is required for early detection and management of associated malignancies.

## Abbreviations

ADHD: Attention deficit hyperactivity disorder
CMMR-D: Constitutional mismatch repair deficiency
CRC: Colorectal cancer
CT scan: Computed tomography scan
GERD: Gastro-esophageal reflux disease
H&E: Hematoxylin and eosin tissue staining
HNPCC: Hereditary non-polyposis colon cancer
LS: Lynch syndrome
*MMR*: Mismatch repair
MSI: Microsatellite instability
SBO: Small bowel obstruction
US: United States of America
